# Regional disparity of covid-19 infections: an investigation using state-level Indian data

**DOI:** 10.1007/s41775-021-00113-w

**Published:** 2021-05-27

**Authors:** Parantap Basu, Ritwik Mazumder

**Affiliations:** 1 Durham University Business School, Durham, UK; 2 Assam University, Silchar, India

**Keywords:** Covid-19 infections, Covid testing, Herd immunity, Urbanization, Poverty, I18, I31

## Abstract

Using the state-level panel data for India, we establish that Covid infections are clustered in more urbanized, and prosperous states. Poverty lowers cases showing evidence of herd immunity of poor which stands in sharp contrast with the developed part of the world. Our dynamic panel regression results indicate that Covid infections are persistent across states and unlocking has aggravated the infections. We also find that richer and more urbanised states with better health infrastructure and governance perform more tests. The policy lesson from this exercise is that the authorities should monitor immunization and Covid protocols in densely populated urban areas.

## Introduction

The vicious Covid-19 virus has its spectre all over the world for over a year now. There is a wave of research about various aspects of this infections which include dynamics of disease spread, (SIR models), the effect of social distancing on infections, the economic effects of Covid lock down amongst others.^1^ However, scarce efforts are devoted to understand the regional disparity in infections and what particularly drives the cluster of infections in a country. In this paper, we explore this issue using India as a testbed.

India is chosen as our case study particularly because of its wide regional diversity in several dimensions. In terms of land area, it is the 7th largest country in the world and it is the home to almost 1.38 billion people (second largest population after China). India is geographically and culturally diverse. Currently with 28 states and 8 centrally administered Union Territories, India speaks at least 21 officially recognised languages and practices 8 major religions having numerous sects within each.

A staggering feature of India is its regional economic inequality. While states like Goa, Kerala, Maharashtra, Punjab and Haryana are highly prosperous (along with the Union Territories of Delhi, Chandigarh and Puducherry), the eastern, north eastern and central states are lagging behind in terms of both economic and human development indicators. For instance, Goa had a per capita net state domestic product (NSDP per capita) of Rs. 467,998 at 2011–2012 prices in 2018–2019 followed by Delhi (at Rs. 365,529), Haryana (at Rs. 226,644) and Puducherry (at Rs. 220,461). At the other extreme, Bihar had a current Rupee per capita NSDP of just Rs. 43,822 in the same year which is just 9 per cent of the corresponding figure for Goa.^2^ The discretionary transfers have not only grown as a proportion of revenue sharing with the states in recent years but have favoured states with higher per capita income. This is particularly true for transfers from the Union government that are allocated in accordance with a matching formulae.^3^

In terms of human development indicators the inter-state disparities are perhaps astonishing. Kerala and Goa with near hundred per cent total literacy rates had infant mortality rates of 7 and 8 respectively per 1000 live births in 2016–2017.^4^ These are better figures compared to Turkey (at 8.6), Ukraine (at 7.2), Kazakhstan (at 9.3), Brazil (12.4), Mexico (12.2), Thailand (at 7.7) and Malaysia (at 7.3). The corresponding figures for Bihar, Madhya Pradesh and Rajasthan stood at 38, 47 and 41, respectively, which is worse than Kenya at 34, Tanzania at 36, and Uganda with 44.7 (in 2019–2020 according to the World Bank). Coming to literacy, with just 70.9% total literacy rate, Bihar is 6.8% below the national average total literacy rate of 77.7%. Total literacy-wise Andhra Pradesh is currently at the bottom with 66.4%, followed by Rajasthan at 69.7%.

In terms of Covid infections, India is currently ranked as one of the leading Covid-19 epicentres in the world in terms of the aggregate number of infections. Since September 15, 2020, this virus is on a decelerating trend but it has taken a nasty turn particularly at the time when this paper was completed. It is unlikely that this virus will go away on its own without immunization of at least 60 per cent of the nation’s population. The good news is that alternative vaccines are in place but it is uncertain whether it will reach all the vulnerable belts of the country within a reasonable deadline. Our study covers the pandemic period until February 21 and has nothing to say about the present massive spikes in cases and fatalities. The key results and the policy conclusion that we reach may thus be tentative.

We use the state-level cross section as well panel data to identify the possible clusters of Covid infections in India. Our data source is the real-time database available in www.covid19india.org. To the best of our knowledge, this is the most comprehensive dataset for Covid infections in different regions of India which are widely used by researchers.

Two key findings come out of our investigation. First, Covid infections are clustered in the rich and industrial states with a high population density. Agricultural regions have fewer infections. Since tests can only detect infections, we find that more tests are undertaken in relatively prosperous regions which is indicative of uneven development and lack of governance in poorer regions. This is confirmed by the fact that tests are lower in states where there is inferior law and order and higher infant mortality.

The second robust finding from our study is that infections are uniformly lower in regions with greater poverty. This is true for both urban and agricultural states. This result stands in sharp contrast with the experience of the developed hemisphere including United States. Our tentative hypothesis for this difference in response of infections to poverty is the *hygiene hypothesis* well known in the epidemiology literature that poor in developing country like India have acquired herd immunity due to exposure to different types of infections from childhood.

Our paper relates to a fast growing literature on the effect of poverty and inequality on the incidence of pandemic. There is no robust convincing evidence that inequality has a negative effect on health although inequality when combined with poverty may have detrimental effect on health due to unequal health care services (Deaton, 2013). In the United States, black, Hispanic and indigenous population were exposed more to Covid infections than whites (Hoyer and Morrison, 2020). Using preliminary US county level analysis, Abedi et al. (2020) document that existing rates of poverty, disease and the presence of ethnic minorities were all associated with higher infection. UK also has similar experiences among black, Asian and Middle Eastern (known as BAME) groups (Office of National Statistics, 2020). Finch and Hernández Finch (2020) find that more disadvantaged counties in the United States had a larger number of confirmed Covid-19 cases, and that the number of Covid-19-related deaths was associated with poorer and more urban counties. Quite remarkably, they observe that the testing for the virus was less available in more disadvantaged counties. Viewed from this perspective, the experience of India in terms of the relationship between poverty and cases is unique. Our study parallels Davies (2021) who undertakes cross country analysis of Covid-19 death rates and finds that poverty has a weak negative association with case fatalities. Our focus is, however, on cases and not on fatalities.^5^

The paper is organized as follows. In Sect. 2, we present a brief literature review of the extant studies on Covid 19 in India. In Sect. 3, we report the main pockets of infections in India and point to some possible developmental covariates of infections. In Sect. 4, we develop a simple SIR model to put some structure to our regression analysis and report cross sectional regressions of the possible determinants of infections with a focus on development indicators in each state. In Sect. 5, we undertake panel regressions to understand the dynamics of infections and the effect of removing lockdown restrictions. Section 6 addresses the question why richer states in India are testing more. Section 7 explores the relationship between poverty and infections. Section 8 concludes.

## Literature on Covid-19 in India

A number of recent studies have examined the trend in Covid-19 infections in India and its regional disparity. Jalan and Sen (2020a) were amongst the first to systematically observe on the basis of district level data till the midnight of April 5, 2020, that not all of India had been impacted uniformly by the virus, and that there was a strong case in favour of implementation of a more selective lockdown. The heavily affected districts included the metro areas of Delhi, Mumbai, Indore, Jaipur, Chennai, and Pune. The 20 significantly affected districts included Agra, Ahmedabad, Bengaluru, Coimbatore, and Thane while 42 districts were moderately affected, and 188 districts were mildly affected. The unevenness in the spread of the virus was understood by the fact that Jammu and Kashmir, Telangana, and an additional 16 per cent of all the districts in the rest of India together reported 86 per cent of all Covid cases in the country.^6^ In a later study for the state of Kerala, Jalan and Sen (2020b) found that the state effectively managed its first Covid wave by formulating a comprehensive set of government actions that were supported and complied by the state’s citizens. The authors maintain that this was achieved by leveraging and reinforcing the citizen’s public trust in the state. The regional disparity in COVID infections in India has also been observed by Mandi et al. (2020). They constructed a multi-dimensional vulnerability Index for Indian districts with an aim to provide a direction for sequential lifting of the lockdown. Ray and Subramanian (2020) also noted the regional disparity in infections although their key objective was to provide an interim report on the Indian lockdown provoked by the COVID-19 pandemic.

Our central research question is: how do socioeconomic and macro development indicators explain infection differentials across Indian states? This key question largely remains unanswered in the extant studies. Moreover, these papers hardly undertake an econometric investigation of the macro developmental determinants of the regional disparity of cases in India during the first wave of this pandemic. In this respect, our study is novel.

## Where are infections and tests in India?

Our observations based on recent state-level panel data on Covid statistics reveal several important empirical regularities. Data for total confirmed cases and tests conducted per million populations till 21-01-2021 are only considered across 33 states and union territories, leaving out extreme outliers like Ladakh and Lakshadweep.^7^ The remaining state-level macroeconomic data are drawn from various secondary sources listed in the appendix.

**Fig. 1:**
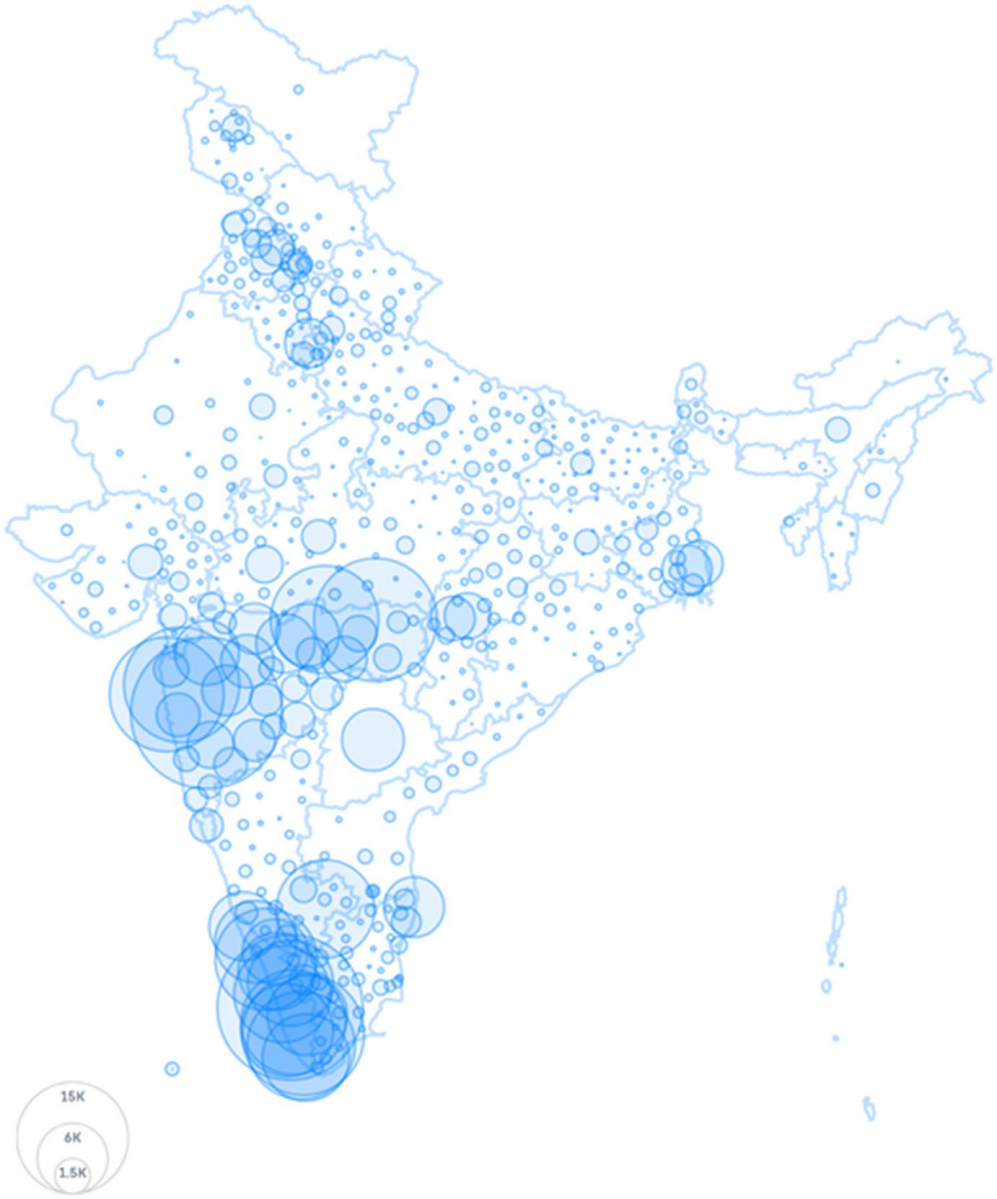
India’s Covid-19 clusters showing spatial distribution of confirmed cases. Confirmed Covid positive cases per million state populations are for 21-02-2021. Source: https://www.covid19india.org

**Fig. 2:**
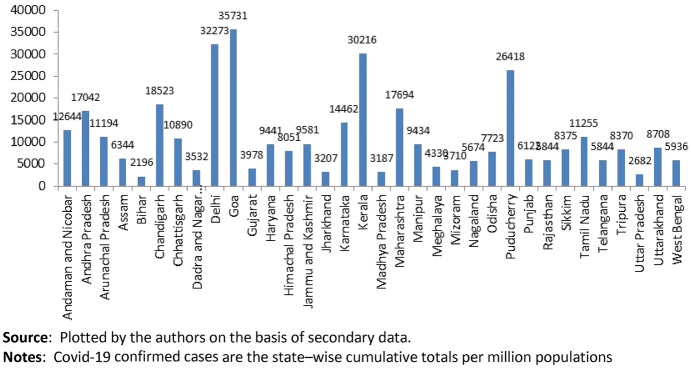
State-wise Covid-19 confirmed cases per million population (as on March 01, 2021)

The spatial distribution of Covid-positive cases per million can be visualised from India’s February 21, 2021 Covid-19 infections map as shown in Fig. 1. The colour patch concentrations are clearly indicative of cluster of Covid infections in and around the major urban and developed centres of India—namely, Mumbai and its surroundings (in Maharashtra), Kerala, Chennai (and its neighbouring areas), Delhi National Capital Region, Chandigarh and other major urban locations in Northern and Central India along with Kolkata and its surrounding districts in West Bengal. The bar chart in Fig. 2 provides more details about the cross state variation of infections. Infections stand out in Delhi, Goa, Kerala, Maharashtra, and Puducherry.

**Fig. 3:**
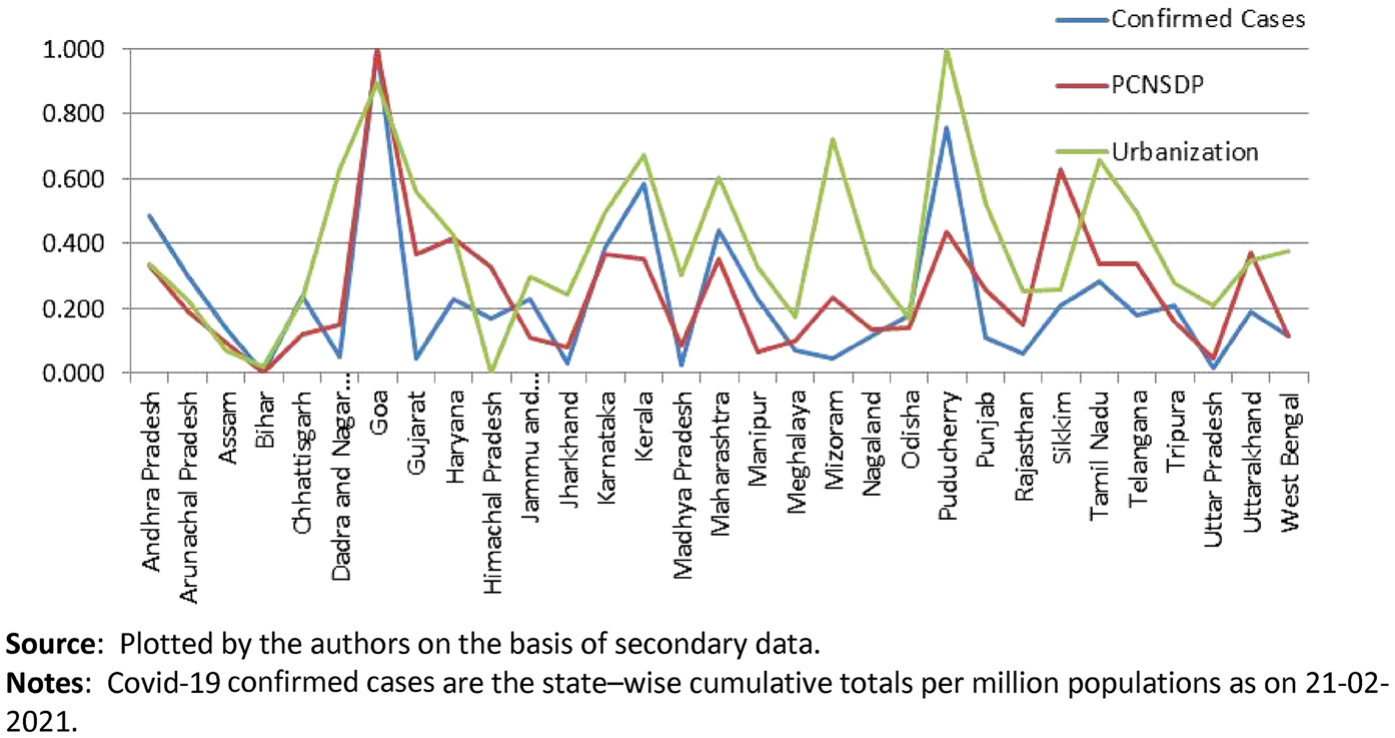
Comparing confirmed cases, per captia NSDP and the level of Urbanisation across Indian States

Probing further to understand how infections associate with various developmental indicators, we find that the incidence of this virus (in terms of confirmed cases per million state populations) is significantly more in prosperous states in terms per capita net domestic product (PCNSDP) and with higher degrees of urbanization. This is shown in Fig. 3.^8^ We capture urbanization with the help of percentage of urban population at the state level (as per Census 2011 figures). Higher levels of urbanization and economic development invariably result in higher population densities at the state level especially because urbanized states attract migrant workers and people from backward and poorer states.

**Fig. 4:**
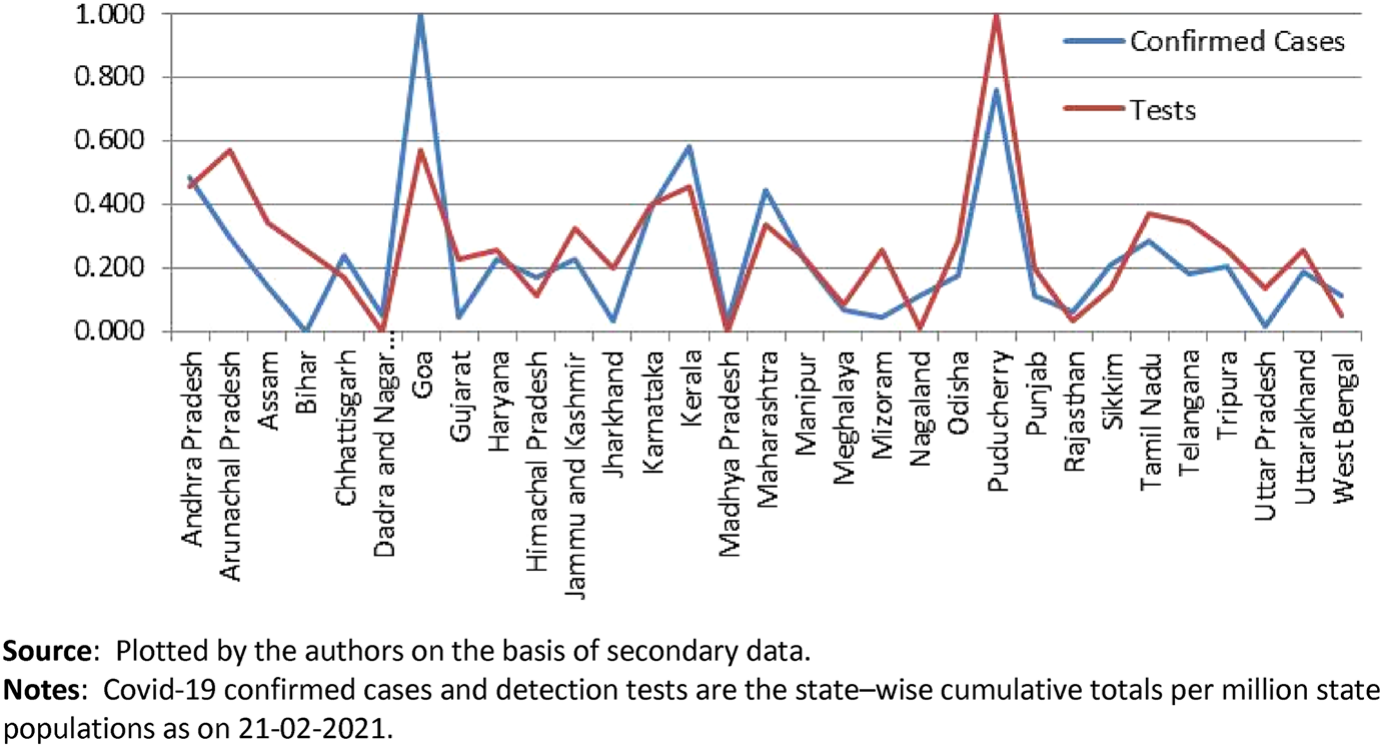
Comparing Confirmed cases, per million and tests conducted per million across Indian states. Covid-19 confirmed cases and detection tests are the state-wise cumulative totals per million state populations as on 21-02-2021

Not surprisingly, there is a close association between state-level tests per million and reported cases per million as seen in Fig. 4. The correlation coefficient between tests and cases is 0.65 and is statistically highly significant (see Table 1).

**Table 1:** Bi-variate ordinary correlations between variables of Interest

Variables	CASES	TESTS	PCNSDP	URBAN	DENSITY	BPL	AGRI
CASES	1						
TESTS	0.65 (0.000)	1					
PCNSDP	0.77 (0.000)	0.35 (0.041)	1				
URBAN	0.78 (0.000)	0.49 (0.003)	0.68 (0.000)	1			
DENSITY	0.57 (0.000)	0.213 (0.000)	0.37 (0.033)	0.723 (0.000)	1		
BPL	− 0.37 (0.033)	− 0.31 (0.069)	− 0.51 (0.002)	− 0.35 (0.043)	− 0.22 (0.244)	1	
AGRI	− 0.77 (0.000)	− 0.54 (0.001)	− 0.72 (0.000)	− 0.69 (0.000)	− 0.59 (0.000)	0.34 (0.050)	1

A cross-section of 33 states and Union Territories in India are taken for computing these correlations

Source: Estimated by the authors on the basis of secondary data

*P* values are given in parenthesis

The larger question is: which states are testing more? A part of it is answered in Fig. 4 itself which shows that more urbanised states are testing more and as a result getting more confirmed cases reported. The degree of state-level urbanization may be taken as an effective proxy for the level of economic development in the Indian context.

Table 1 presents a few key bivariate correlation coefficients. Few points are worthy of attention. First, cases (CASES) are strongly associated with tests (TESTS). More prosperous, urban and densely populated states with better infrastructure report more infections. Tests are happening more in developed states with better infrastructure. Agricultural share in state GDP (AGRI) is negatively associated with both confirmed cases and tests. Moreover agriculturally dominant states tend to have lower population density (DENSITY) as well as lower levels of urbanization (URBAN).

Second, poverty measured by percent people below poverty line (BPL) has a significant negative association with confirmed cases per million which indicates that poorer states tend to have lower infections per million. This comes as a surprise because it goes against a few extant studies based on UK and US which find poverty as a driver of infections due to lack of Covid-safe social distancing.^9^ In India also, it is difficult for the low income citizens to maintain social distancing besides maintaining the desirable standards of personal health, hygiene and sanitation. However, it should also be borne in mind that poorer states get tested less as evidenced by a negative correlation between cases and BPL.

## Role of development and poverty in determining infections

Motivated by the correlation matrix in Table 1 and the spatial distribution map of infections, we next explore the structural determinants of infections. To fix ideas, we start off with a simple discretised version of a SIR model formulated by Kermack and McKendrick (1927). Population normalized at unity is divided in three distinct groups at date t, (i) susceptible $\left( {S_{t} } \right)$, (ii) infected $\left( {I_{t} } \right)$ and (iii) recovered $\left( {R_{t} } \right)$ where $S_{t} + I_{t} + R_{t} = 1$.^10^ The rate of new infections is $\beta {S}_{t}{I}_{t}$ if infected and susceptible meet at a contact rate $\beta$. The rate of recovery is *γ*.

The evolution of SIR population is given by the relationship as follows:

1 $$\Delta I_{t} = \beta S_{t} I_{t} - \gamma I_{t} - \delta A_{t}$$

where $\Delta I_{t} = I_{t + 1} - I_{t}$ with *γ* ∈ (0,1), $\delta >0$. ${A}_{t}$ is a public health policy variable which depends on the quality of health care and availability of an efficacious vaccine. Everything else equal, the higher the ${A}_{t}$, the lower the infections. The effectiveness of the policy in lowering infection is characterized by the parameter $\delta$.

We add a social distancing dimension to this SIR model. The contact between $${S}_{t}$$ and $${I}_{t}$$ groups is determined by social distancing ($${D}_{t})$$. The higher the $${D}_{t}$$, the lower the contact $${S}_{t}{I}_{t}.$$ Let us posit:

2 $$S_{t} I_{t} = c - \lambda D_{t} \;{\text{with}}\;\lambda { > 0}$$

where c is a positive constant. Combining (1) and (2), we get a simple reduced form infection equation:

3 $$I_{t + 1} = \beta c + (1 - \gamma )I_{t} - \beta \lambda D_{t} - \delta A_{t}$$

The left hand side of (3) is the cumulative infection which depends inversely on social distancing. The social distancing ($${D}_{t})$$ is a behavioural variable which depends on a range of developmental and policy variables including lockdown.

The steady state timeless version of (3) is:

4 $$I = a_{0} - a_{1} D - a_{2} A$$

where $$a_{0} = \beta c\gamma^{ - 1} ,\;a_{1} = \beta \lambda \gamma^{ - 1} \;{\text{and}}\;a_{2} = \delta \gamma^{ - 1}$$.

Motivated by the reduced form infection Eq. (4), we first run a log-linear cross-state regressions in Table 2 to focus on various developmental determinants of infections across India. We choose PCNSDP, URBAN, DENSITY, BPL and AGRI as explanatory developmental variables.

**Table 2:** Log-linear regression of confirmed cases per million for 33 states and Union Territories in India

Dependent variable: Log(Cases)	Model 1	Model 2	Model 3	Model 4	Model 5
Explanatory variables					
Constant	9.170**(12.652)	8.750**(12.003)	8.739**(11.860)	2.541(1.054)	10.872** (27.839)
Log(PCNSDP)				0.618**(3.262)	0.190**(2.870)
Log(URBAN)	0.324*(2.272)	0.443**(2.860)	0.335*(2.329)		
Log(AGRI)	− 0.371*(− 3.489)	− 0.372**(− 3.809)	− 0.243(− 1.773)	− 0.277*(− 2.322)	
Log(BPL)	− 0.155*(− 2.200)				
Log(WFA)					− 0.363**(− 4.928)
Log(URBAN)*Log(BPL)		− 0.044*(− 2.183)			
R square	0.59	0.59	0.58	0.66	0.66
Adjusted R square	0.56	055	0.54	0.64	0.63
F statistics	14.75**	14.73**	14.05**	29.79**	28.90**
Durbin-Watson	2.15	2.16	2.12	1.99	2.12

Source: Estimated by the authors on the basis of secondary data

(1) Numbers in the parentheses are t ratios where HAC adjusted standard errors are used in all cases. **means significant at 1% level and **means significant at 5% level. (2) The total state level confirmed cases and tests per million population are for 21-02-2021

Few observations in Table 2 lend themselves for attention*.* First, level of urbanization and PCNSDP have significant positive influence over infections—a robust finding across all specifications. Second, the agricultural share in state GDP has a dampening effect on confirmed cases. In other words, agrarian states tend to have lower infections. Third, BPL has a negative effect on infections significant at the 5% level. When BPL is interacted with URBAN, we find that it mutes the positive effect of urbanization on infections. This observation points to a potent testable hypothesis that people below poverty line living in more dense and urban areas may be resilient to this nasty virus. In other words, there is a nonlinear interaction between poverty and urbanization in determining infections.^11^

In a nutshell, this cross section regression suggests that Covid infections are highly concentrated in more prosperous, urbanized (i.e., relatively more developed) and densely populated states. Poverty lowers infections and it mutes the positive effects of urbanization on infections. Agrarian states in India have lower population density and thus lower levels of urbanizations leading to lower infections per million. The percentage of work force engaged in agriculture (WFA) in the total labour force introduced in Model 5 adds new insights that explain the limited spread of the covid in agriculturally dominant states. Controlling for per capita NSDP, WFA shows a significantly negative influence over confirmed cases, implying that when income is controlled higher proportions of workers engaged in agriculture and allied activities control the spread of the virus to a significant extent. For instance, Bihar, Chhattisgarh and Madhya Pradesh, each with more than 70% work force engaged in agriculture and allied activities are amongst the lowest Covid-19 affected in the country.

WFA is significantly negatively associated with urbanization as seen in correlations in Table 5. The chance of Covid transmission is significantly lower in states with a higher agricultural dominance (measured both in terms of WFA and AGRI) due to lower population density. On the other hand, more urbanised states, (which are also states with greater proportion of formal sector or urban sector workers) have greater potentials of Covid-19 spread due to the failure to maintain ideal social distancing norms, as ideal social distancing practices in India are difficult to maintain in a fast and unrestricted urban life. In a nutshell, the poor and backward agrarian states may be practicing what may be called “*natural social distancing*” due to lower population density which explains less infections in these regions.

## Dynamic panel regression

We next run a dynamic panel regression motivated by (3) involving the same 33 states and UTs weekly data starting March 2020 till February 21, 2021 (47 weekly observations for each state yielding 1551 pooled observation). The results shown in Table 3 reinforce the observations of the cross-section regressions of Table 2 and give additional insights. A one period lag of log of cases is introduced throughout to eliminate serial correlation in accordance with SIR Eq. (3). In addition, we introduce an “unlock” dummy (UNLOCK) that takes the value unity for post July 1, 2020 observations and 0 otherwise, to demarcate the pre and post unlock 2 phases in India. The sign of the “unlock” dummy will capture the effect of less policy stringency of social distancing on infections.

**Table 3:** The Log-linear panel regression of weekly cumulative total confirmed cases on state-level factors [Depended variable: Log(Cases)]

Explanatory variables	Model 1	Model 2	Model 3	Model 4
Constant	0.808**(4.184)	1.264**(7.971)	1.175**(4.109)	− 0.873(− 1.880)
Log(Cases(− 1))	0.824**(34.362)	0.873**(58.032)	0.825**(24.882)	0.872**(50.713)
Log(PCNSDP)				0.129**(3.125)
Log(URBAN)	0.136*(2.032)		0.156**(2.582)	
Log(DENSITY)	0.041*(2.125)			0.049**(2.653)
Log(BPL)	− 0.064*(− 2.002)	− 0.062**(− 3.034)		
Log(AGRI)		− 0.080**(− 4.776)	− 0.068*(− 2.040)	
Unlock		0.244**(3.059)		0.258**(3.317)
Log(URBAN)*Log(BPL)			− 0.023*(− 2.153)	
R-squared	0.96	0.96	0.96	0.96
Adjusted R-squared	0.96	0.96	0.96	0.96
F-statistic	10480.21**	10481.78**	10481.65**	10518.06

Source: Estimated by the authors on the basis of secondary data

(1) Numbers in the parentheses are *t* ratios where White’s diagonally corrected standard errors are used throughout. **means significant at 1% level and *means significant at 5% level. (2) These pooled estimates use White’s diagonally corrected standard errors throughout. (3) Number of states and UTs = 33, number of weeks = 47; panel includes 1551 pooled observations

Few important observations are in order. First, the significantly high lagged coefficient of regression is indicative of the persistent nature of infections. One per cent rise in infections in the previous week is associated with at least 0.88 percent increase infections in the current week. Second, the “unlock” phase has significantly added to Covid confirmed numbers at the state level as seen in models 2 to 4 ‘UNLOCK coefficient is positive and significant. Finally, other things unchanged, higher poverty rates at the state level lead to uniformly lower infections which is a consistent finding across both cross-section and panel regression models.

Finally we repeat our panel estimation of a similar family of log-linear regression models (see Table 4) but this time with the log differenced confirmed cases per million state population (which captures the weekly new cases per million) as the dependent variable. To capture the nonlinearity of the weekly count curve over time during March 2020 to February 2021, we insert time and time-squared as regressors and drop the ‘unlock’ dummy. Even though differencing introduces greater variability in the data, it is noteworthy that the negative BPL coefficient is still significant at the 10% level. The results are overall consistent with Tables 2 and 3. Time has a significantly positive coefficient and time-squared has a negative coefficient. In other words, new infections show an inverted U-shaped pattern meaning that it peaks and then dies out. The one period lagged coefficient is also akin to the previous regressions showing persistence in new infections and this pattern is consistent across all these four models.

**Table 4:** The Log-linear panel regression of weekly new confirmed cases on state-level factors [Depended variable: log(D(cases))]

Explanatory variables	Model 1	Model 2	Model 3	Model 4
Constant	− 0.492(− 0.911)	− 0.467(− 1.691)	0.816*(2.110)	− 3.560**(− 4.412)
Log(D(Cases(− 1))	0.785**(34.931)	0.796**(23.556)	0.774**(21.010)	0.779**(21.745)
Log(PCNSDP)				0.234**(3.925)
Log(URBAN)	0.149(1.540)		0.156*(2.059)	
Log(DENSITY)		0.070**(2.932)		0.073**(3.005)
Log(AGRI)	− 0.075*(− 2.011)		− 0.079(− 1.834)	
Log(BPL)	− 0.079(− 1.801)	− 0.087(− 1.770)	− 0.083(− 1.724)	
TIME	0.132**(4.919)	0.122**(4.820)		0.138**(5.099)
Time-squared	− 0.002**(− 5.497)	− 0.002**(5.430)		− 0.003**(− 5.668)
R-squared	0.91	0.91	0.91	0.91
Adjusted R-squared	0.91	0.91	0.91	0.91
F-statistic	2539.62**	3027.57**	3041.57**	3062.94**

Source: Estimated by the authors on the basis of secondary data

(1) Numbers in the parentheses are t ratios which use White’s diagonally corrected standard errors throughout. **means significant at 1% level and *means significant at 5% level. (2) Number of states and UTs = 33, number of weeks = 47; panel includes 1551 pooled observations. (3) D(cases) imply the first difference of cumulative weekly total cases, which is tantamount to weekly count per million or weekly new cases per million. (4) Model 3 is estimated under fixed time effects (periods fixed) supressing time and time-squared

## Why are rich states getting tested more?

Since TESTS and PCNSDP show strong positive correlation as seen in Table 1, a natural question arises why are tests so much concentrated in prosperous regions of India? Do these regions have a better health infrastructure and better governance? States which are better governed and have a more robust public health policy are likely to undertake more tests. To this end, we take infant mortality rate (IMR) as a proxy for the adequacy of the public health infrastructure. In the Indian context, IMR is taken as a proxy for the state-level backwardness in terms of general health status and health infrastructure. Thus regions with lower infant mortality rate are likely to have a better health infrastructure and arguably better health status.

Regarding governance, we pick the total crime rate (TCR) as a proxy for governance keeping in mind that TCR reflects reported and government recorded crime. Crimes reported but unregistered by officials are not included in TCR. In our view, better governance leads to more reported and registered crimes and so better governance may be reflected in higher TCR.^12^

The correlations reported in Table 5 reveal that, high IMR states tend to have low TCR. Furthermore, the fact that URBAN and PCNSDP are positively associated with TCR but negatively with BPL and AGRI implies that while richer and developed states report and register more crimes, poorer states and states with higher AGRI do not. These are supportive of our idea of taking TCR as a proxy for governance. Strong positive correlations of TCR with PCNSDP and URBAN suggests that developed states in India tend to report more crimes and thus have relatively better standards of governance.

**Table 5:** Selected bi-variate ordinary correlation coefficients

Variables	TCR	IMR	BPL	Urban	PCNSDP	AGRI	WFA
TCR	1						
IMR	− 0.18(0.278)	1					
BPL	− 0.26(0.122)	0.64(0.000)	1				
URBAN	0.57(0.000)	− 0.44(0.011)	− 0.35(0.043)	1			
PCNSDP	0.33(0.066)	− 0.51(0.001)	− 0.51(0.002)	0.68(0.000)	1		
AGRI	− 0.39(0.072)	0.63(0.000)	0.34(0.049)	− 0.69(0.000)	− 0.72(0.000)	1	
WFA	− 0.52(0.002)	0.66(0.000)	0.46(0.007)	− 0.85(0.000)	− 0.64(0.000)	0.72(0.000)	1

Source: Estimated by the authors on the basis of secondary data

*P* values are shown in parentheses

In addition, the fact that high IMR basically captures state-level backwardness is understood from its significantly negative association with URBAN and PCNSDP (each of which are strong socio-economic development indicators on its own merit) besides its positive association with BPL (and even AGRI). In other words, developed states tend to have low IMR and this gives sufficient justifications for choosing IMR as an indicator of state-level backwardness.

Table 6 presents the results of a dynamic log-linear panel regression of tests with TCR and IMR and URBAN as three variables capturing state-level fixed effects. After controlling for the serial correlation in tests (by addition lagged test variable as a regressor), the state-level infrastructure and governance variables are significant determinants of tests. States with better health and urban infrastructure and better governance carry out more tests which is consistent with the bivariate correlations reported in Table 1.

**Table 6:** Explaining Covid-19 tests in India with the help of governance and development variables [Dependent Variable: Log(tests)]

Variables	Coefficient	SE	t-Stat
Constant	1.471	0.209	7.029**
Log(tests(−1))	0.839	0.008	106.35**
Log(TCR)	0.089	0.018	4.873**
Log(IMR)	− 0.083	0.038	− 2.192*
Log(urban)	0.044	0.021	2.125*
R-square	0.91		
Adjusted R-square	0.91		
F-statistic	3864.10**		

Source: Estimated by the authors on the basis of secondary data

*Implies statistical significance at 5% while **Implies the same at 1%

## Poverty and infections

What is surprising is the response of infections to poverty. Poor people are less susceptible to infections in India which is a robust finding of our study. This finding stands in sharp contrast with the experience of the developed countries, particularly USA and UK where low income essential workers are more affected by the virus compared to the rest of the population. What are the possible reasons for this stark difference in response of infections to poverty in India compared to the developed counterpart of the world? Two hypotheses lend themselves. First, it is possible that poor states are testing less and thus less infections are reported there. This possibility cannot be ruled out given that correlation coefficient between BPL and cases is negative in Table 1 and it is mildly significant statistically.

The second hypothesis is the *hygiene hypothesis* first introduced by the epidemiologist Strachan (1989) who found that children in larger households contracted less hay fever because they are exposed to germs from older siblings. Further research suggested that lack of early childhood exposure to unhygienic environments can make the adults more vulnerable to various kinds of infections.^13^ It is not implausible to hypothesize that in India poor may be immune to various kinds of infections due to unhygienic living conditions from early childhood while in the developed world, the basic health infrastructure permits low income people to access clean and germ free environment from their childhood. Although this hygiene hypothesis is just a conjecture and it needs greater scrutiny, we tend to subscribe to it for the following reasons. We have computed the correlation between case fatalities and the level of development. The correlation coefficient between PCNSDP and FATALITY is a staggering 0.76 which is highly significant implying that the richer states have experienced the highest death rates. In other words, better off people in India died more of Covid cases.

We also have some anecdotal evidence to support this hygiene hypothesis. In India, supply of semi-skilled and unskilled migrant workers engaged in Construction as well as Small and Medium Enterprises (SME) comes from distant rural areas. Under severe distress these migrant workers were forced to leave these urban centres (not just Delhi alone) during the first wave and stringent phase of the lockdowns (i.e., 25th March to 14th April, 2020) resulting in the ‘infamous’ mass urban–rural exodus in India. It is noteworthy that despite this mass movement of migrant workers, the Covid did not spread significantly to poor and backward northern states (mostly Uttar Pradesh, Uttarakhand, Himachal Pradesh, Bihar, Jharkhand, Chhattisgarh, and Madhya Pradesh) from where the migrant workers mostly originate. This event reinforces our claim on herd immunity among the poor sections of the society.

## Policy implications and conclusion

Our study shows that Covid infections have significant regional disparity in India. Infections are more concentrated in prosperous Indian states with higher levels of urbanization coupled with high population density. By default, agrarian states have been affected less on account of lower levels of population density and urbanisation. Second, we find that tests also display significant regional disparity. Prosperous states with a better health infrastructure and governance undertake more tests and this also explains why more infections are reported in these regions. Third, a robust finding is that poverty has a dampening effect on infections which suggests some degree of herd immunity among the poor as well as less tests done in poor states.

Our dynamic panel regressions based on the first wave of the pandemic suggest that infections are persistent and new infections seemed to reach plateau and then started declining. Evidently this dynamics has now reversed with the new wave of infections which is beyond the scope of our study. The unlock phase during the post July 2020, has added significantly to state-wise Covid numbers. The policy lesson that we learn from this exercise is that, the authority should continue to monitor densely populated urban areas without compromising the standard of Covid prevention protocols including the rates of testing in poor, remote and backward Indian states.

Although a couple of efficacious vaccines are already in place, the universally accepted Covid protocols of safe social distancing with mandatory preventive measures as masks and sanitizers are the only means of decelerating the rate of spread of Covid infections. As new official reports about the spread of a more potent and virulent strain of this virus arrive, it is crucially important that crowds and gatherings are controlled and Covid surveillance practices are aggressively stepped up by state governments including punitive actions in all cases of violations. After all, Covid prevention and control is an issue that is inextricably linked with the quality and efficiency of governance at the local levels together with the efficacy of the vaccines.

Our study can be extended further. We have focused on the first wave of infections and only looked at cases not fatalities. Given the present trend in Covid-19 now, it is important to explore the dynamics and regional variation of case fatalities in India. Such a study also requires careful modelling of multiple Covid cycles using the SIR framework.

## Appendix

### Variable definitions and data sources

AGRI—Percentage contribution of State Domestic Product from agriculture and allied activities, compiled from RBI Handbook of Statistics on Indian Economy available at https://www.rbi.org.in/scripts/AnnualPublications.aspx? (Table 8: Net State Value Added by Economic Activity at Constant Prices, Base: 2011–2012).

BPL—Percentage of population below poverty line at the state level (2011–2012) based on Tendulkar Methodology. State-level figures for combined poverty estimates were obtained from https://niti.gov.in/state-statistics (Data Source: Planning Commission).

CASES—confirmed cumulative total covid-19 Infections per million state populations as on 14/02/2021. (Source: https://www.covid19india.org for India.

DENSITY—Population density per sq.km as per 2011 Census, compiled from https://www.census2011.co.in/density.php for India (Source: Census of India, 2011), and 2010.

GINI—State Wise Gini Coefficient for Household Asset Scores, NFHS -4, 2015–2016 (taken from Pandey & Gautam, 2020, Table 1, pp. 23).

IMR**—**Infant Mortality Rate (per 1000 live births) for 2016 obtained from the Niti Ayog, Government of India, available at, https://niti.gov.in/content/infant-mortality-rate-imr-1000-live-births (Source: Sample Registration System).

PCNSDP—Per capita NSDP for 2018–2019, at 2011–2012 prices, compiled from RBI Handbook of Statistics on Indian Economy available at https://www.rbi.org.in/scripts/PublicationsView.aspx?id=19743. [Source: National Statistical Office (NSO)].

TESTS—Cumulative total Tests conducted per million at the state level as on 21/02/2021(Source: https://www.covid19india.org).

TCR—Total Crime Rate (reported) compiled from ‘Crime in India 2018’, National Crime Records Bureau (Ministry of Home Affairs, Government of India), page 9, Table 1. A.1 IPC Crimes (State/UT-wise)—2016–2018 available at https://ncrb.gov.in/sites/default/files/Crime.

URBAN—urban population as a percentage of state population based on 2011 Census. For each state it is compiled from https://www.census2011.co.in/census/state/ (Source: Census of India, 2011).

WFA—Work force in Agriculture and allied activities expressed as percentage of total work force at the state level. Source: Census of India 2011, Table T 00-009: Distribution of workers by category of workers (https://censusindia.gov.in/tables_published/a-series/a-series_links/t_00_009.aspx).

## Footnotes

1 For a comprehensive real time papers on Covid, see Covid Economics CEPR volume, https://cepr.org/content/covid-economics-vetted-and-real-time-papers-0.

2 Source: Central Statistical Office, Ministry of Statistics and Programme Implementation, Government of India.

3 See Rao and Singh 2006, Kletzer and Singh 1997, Chelliah et al. (1992) for a detailed overview.

4 *Niti Ayog*, Government of India.

5 There is also a growing stream literature on voluntary social distancing of poor. Brown and Ravallion (2020) undertake a comprehensive survey of 3000 US counties and find that social distancing is costly for the poor. Brotherhood et al. (2020) also reach similar conclusion focusing on two major Brazilian cities. None of these papers look at the regional variation of and its possible determinants which is the central focus in this paper.

6 For a discussion on the methodology behind categorization of districts into heavily affected, significantly affected, moderately affected and mildly affected, see Jalan and Sen (2020a, available at https://theprint.in/opinion/understand-the-method-in-covid-19s-madness-india-doesnt-need-complete-lockdown/398925/ and also in *The Wire,* May 5, 2020).

7 Cut-off from India’s mainland, the union territories of Lakshadweep and Ladakh are dropped as outliers in terms of Covid-19 infections and population density. Data on several variables of interest for the UTs of Daman and Diu as well as Dadra and Nagar Haveli are unavailable.

8 For line graph plotting, variables are normalized to a (0, 1) scale for each state by means of a UNDP-HDI type attainment index formula to facilitate comparison. This is simply the difference of the actual value from the minimum in the sample, divided by the range in the sample (i.e., maximum minus minimum values).

9 Basu et al. (2021) focus on 83 districts of England and find that safe social distancing is costly for low income households. Similar results are reached by Brown and Ravallion (2020) covering 3000 counties of the US.

10 Since our focus is on Covid infections, we ignore deaths from this model.

11 Similar nonlinearity is also found in Basu et al. (2021) who find that people socially distance less than rich in poorer and more densely populated districts in England.

12 Underreporting of crime is quite pervasive in India. A state-wise analysis based on National Family Health Survey (NFHS) reports that underreporting of violence against women is higher in states such as Bihar, Uttar Pradesh and Jharkhand which are among the backward state sin India. See https://www.livemint.com/Politics/AV3sIKoEBAGZozALMX8THK/99-cases-of-sexual-assaults-go-unreported-govt-data-shows.html

13 See also https://www.livescience.com/54078-hygiene-hypothesis.html
